# Differences in Gray Matter Volume in Cerebral Small Vessel Disease Patients with and without Sleep Disturbance

**DOI:** 10.3390/brainsci13020294

**Published:** 2023-02-09

**Authors:** Jing Zhao, Qianqian Kong, Xirui Zhou, Yi Zhang, Zhiyuan Yu, Wensheng Qu, Hao Huang, Xiang Luo

**Affiliations:** Department of Neurology, Tongji Hospital, Tongji Medical College, Huazhong University of Science and Technology, Wuhan 430022, China

**Keywords:** cerebral small vessel disease, sleep disturbance, gray matter volume, white matter hyperintensities, magnetic resonance imaging

## Abstract

Recently, there has been increased interest in the relationship between cerebral small vessel disease (CSVD) and circadian rhythm disruption, particularly sleep disturbance. However, the neural mechanism of sleep disturbance in CSVD patients remains poorly understood. The purpose of this study is to explore the gray matter alterations in CSVD patients with and without sleep disturbance. 59 patients with CSVD and 40 healthy controls (HC) were recruited for the present study. Sleep quality was assessed using the Pittsburgh Sleep Quality Index (PSQI) questionnaire. CSVD patients were categorized into either the good sleepers group (CSVD-GS, *n* = 23) or the poor sleepers group (CSVD-PS, *n* = 36) based on PSQI score. Voxel-based morphometry (VBM) analysis was used to assess differences in gray matter volume (GMV) between groups. Multivariate regression analyses were performed to investigate the relationships between sleep quality, GMV, and white matter hyperintensities (WMH). We observed GMV differences between the three groups in the bilateral caudate, right thalamus, bilateral calcarine cortex, left precentral gyrus, right orbitofrontal cortex, left cingulate gyrus, and right sub-gyral temporal lobe. Additionally, the CSVD-PS group exhibited decreased GMV in the bilateral calcarine cortex yet increased GMV in the right caudate compared to the CSVD-GS group. In fully adjusted models, GMV of the right caudate and bilateral calcarine cortex was associated with sleep quality in CSVD patients. The present study revealed structural brain alterations in CSVD patients with sleep disturbance. These findings may provide novel insights into the neural mechanisms of sleep disturbance in CSVD.

## 1. Introduction

Cerebral small vessel disease (CSVD) is a progressive syndrome related to the small vessels of the brain and manifests as white matter hyperintensities (WMH), lacunes, enlarged perivascular spaces (EPVS), cerebral microbleeds (CMBs), and atrophy on conventional brain magnetic resonance imaging (MRI) [1,2]. The incidence of CSVD increases with age [3]. Thus, with the aging population, the clinical symptoms caused by CSVD have received more attention. Recently, in addition to stroke, cognitive decline, gait disturbance, and mood disorders, growing evidence also suggests that CSVD is associated with sleep disorders [4,5,6,7,8].

Sleep is increasingly recognized as an important factor in overall health and well-being. Sleep disturbance is common among older adults [9]. Poor sleep can profoundly impact an individual’s quality of life and negatively influence cognition and emotion regulation [10,11]. A study by Wang et al. demonstrated that insomnia in older adults with CSVD is higher than that among older adults in general [5]. In addition, community-based studies have shown that WMH and EPVS were associated with self-reported poor sleep quality [6,12,13]. However, the underlying neural mechanisms of the relationship between poor sleep quality and CSVD remain poorly understood.

The advent and development of MRI techniques have provided a safe, non-invasive tool for investigating brain structure and function [14]. Voxel-based morphometry (VBM) is a widely used automated technique that examines regional brain volume on a voxel-wise basis and may potentially identify subtle structural abnormalities which appear normal on conventional MRI [15]. Utilizing VBM may improve understanding of the brain pathophysiology of the relationship between CSVD and poor sleep quality.

The present study aimed to explore gray matter structural differences between CSVD patients with and without sleep disturbance using VBM analysis. We hypothesized that CSVD patients with poor sleep quality would exhibit alterations in brain structure (gray matter atrophy or hypertrophy) compared to CSVD patients with good sleep quality and that alterations in regional gray matter volume (GMV) would be associated with sleep quality.

## 2. Methods

### 2.1. Participants

**Figure 1:**
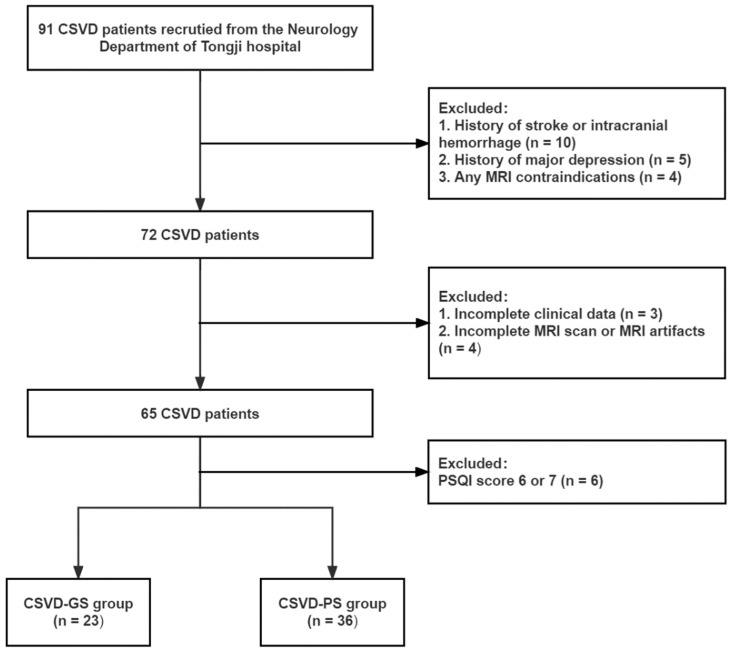
Flowchart of CSVD patient selection. CSVD—cerebral small vessel disease; MRI—magnetic resonance imaging; PSQI—Pittsburgh Sleep Quality Index; PS—poor sleepers; GS—good sleepers.

From January 2019 to February 2021, subjects with CSVD were prospectively and consecutively recruited from the Department of Neurology at Tongji Hospital from outpatient and inpatient services. Subjects were initially referred to the neurology department because of dizziness, headaches, memory impairment, sleep disturbance, or gait instability. The flowchart of CSVD patients is shown in Figure 1. In addition, forty age and sex-matched healthy controls (HC) were also recruited. All participants were right-handed Han Chinese.

The inclusion criteria for CSVD patients were as follows: (1) age ≥ 50 years, (2) moderate to severe WMH, with or without the presence of lacunes [16,17]. The exclusion criteria were as follows: (1) left-handedness, (2) stroke, (3) WMH of nonvascular origin (e.g., multiple sclerosis, poisoning, infection, encephalitis), (4) neurodegenerative diseases (e.g., Alzheimer’s disease, Parkinson’s disease), (5) psychiatric disease (e.g., major depression, schizophrenia), (6) MRI contraindications.

Participants completed the Pittsburgh Sleep Quality Index (PSQI) questionnaire, a self-report questionnaire that assesses the quality of sleep over the past month [18]. A global PSQI score greater than 5 indicates poor sleep quality. In order to improve the validity of poor sleep quality assessment, 6 CSVD patients with PSQI scores of 6 or 7 were classified as borderline cases and excluded from all analyses [19]. Finally, CSVD patients were divided into good sleepers (GS) (PSQI ≤ 5, *n* = 23) and poor sleepers (PS) (PSQI ≥ 8, *n* = 36) subgroups according to their PSQI score. Depressive symptoms were measured using the Hamilton Depression Rating Scale (HAMD) [20]. Medical history collection and evaluation questionnaires were completed within 3 days of MRI acquisition.

This study was approved by the Institutional Review Board of Tongji Hospital, Tongji Medical College, Huazhong University of Science and Technology; the ethical approval code of the study is No. 2019-S105. All participants gave written informed consent in accordance with the Declaration of Helsinki.

### 2.2. MRI Acquisition

Brain MRI was obtained using a single 3T MRI scanner (Discovery MR750, GE Healthcare, Milwaukee, WI, USA) with a 32-channel head coil. The scan protocol includes T1-weighted, T2-weighted, fluid-attenuated inversion recovery (FLAIR), diffusion-weighted imaging (DWI), susceptibility-weighted imaging (SWI), and brain volume (BRAVO) sequences. The scanning parameters of the FLAIR images were as follows: repetition time (TR) = 8425 ms, echo time (TE) = 166 ms, inversion time (TI) = 2100 ms, flip angle (FA) = 111°, matrix = 256 × 256, field of view (FOV) = 220 × 220 mm^2^, slice thickness = 3 mm. The scanning parameters of the high-resolution anatomical T1-weighted images were as follows: TR = 8.3 ms, TE = 3.2 ms, TI = 450 ms, FA = 12°, matrix = 256 × 256, FOV = 256 × 256 mm^2^, slice thickness = 1 mm, number of slices = 188, and voxel size = 1 mm × 1 mm × 1 mm.

### 2.3. Assessment of MRI Markers of CSVD

The MRI markers of CSVD were defined according to the STandards for ReportIng Vascular changes on Euroimaging (STRIVE) criteria [2]. Lacunes were defined as round or ovoid cerebrospinal fluid (CSF) containing cavities with a surrounding rim of hyperintensity in subcortical regions that range from 3 to 15 mm in diameter. The severity of the WMH was assessed using the Fazekas scale [21]. CMBs were defined as rounded hypointense lesions (2–10 mm in diameter) on SWI sequences. EPVS were defined as round, ovoid, or linear lesions, generally smaller than 3 mm in diameter, with a CSF-like signal on all sequences.

### 2.4. MRI Data Processing

The T1 structural images for VBM analysis were processed with the Computational Anatomy Toolbox (CAT12; Structural Brain Mapping group, Jena University Hospital, Jena, Germany; http://www.neuro.uni-jena.de/cat/, accessed on 8 February 2023) within Statistical Parametric Mapping 12 (SPM12; The Wellcome Centre for Human Neuroimaging, London, UK; http://www.fil.ion.ucl.ac.uk/spm/software/spm12/, accessed on 8 February 2023). Prior to pre-processing, all images were manually reoriented to the anterior-posterior commissure. The image pre-processing steps are as follows: All T1-weighted images were spatially normalized to Montreal Neurological Institute (MNI) space, then segmented into gray matter (GM), white matter (WM), and CSF. Subsequently, the gray matter images were modulated using Jacobian determinants. Finally, modulated images were smoothed with an 8 mm full width at half maximum Gaussian kernel. For each individual, total intracranial volume (TIV) was calculated by summing the volumes of GM, WM, and CSF.

WMH volume was manually segmented and calculated from FLAIR images using ITK-SNAP software (University of Pennsylvania, Philadelphia, PA, USA; http://www.itksnap.org/, accessed on 8 February 2023).

### 2.5. Statistical Analysis

Statistical analyses were performed using SPSS 20.0 and SPM12 software. Continuous variables are presented as mean ± standard deviation or median with interquartile range contingent upon whether the data were normally distributed or not normally distributed. Categorical variables are presented as frequency and percentages. Group comparisons of clinical and demographic characteristics were examined using one-way ANOVA or Chi-square tests as appropriate.

For the VBM analysis, one-way ANOVA was completed to determine differences in GMV among the three groups (CSVD-GS, CSVD-PS, and HC groups). Covariates included age, sex, and TIV. The false-discovery rate (FDR; *p* < 0.05) method was used to correct for multiple comparisons with a critical cluster size of 40 voxels. Next, brain regions that showed significant differences among the three groups were selected as regions of interest (ROI), and the mean GMV of each ROI was extracted using MarsBar 0.45 (http://marsbar.sourceforge.net/, accessed on 8 February 2023). Further comparison between groups was analyzed by post hoc *t*-tests (SPSS software, *p* < 0.05, Bonferroni correction).

Finally, multivariate regression analyses were performed to investigate the relationships between PSQI score, GMV differences, and WMH volume in all CSVD patients. WMH volume was log-transformed due to the skewed distribution. The associations were adjusted for potential confounders. Models were initially adjusted for age and sex (model 1). The HAMD scores were significantly different among the three groups; therefore, we further adjusted for depressive symptoms in model 2. Additionally, model 3 was further adjusted for WMH volume.

## 3. Results

### 3.1. Demographic and Clinical Data

The demographic and clinical characteristics of the three groups are summarized in Table 1. There were no significant differences in age, sex, years of education, TIV, and vascular risk factors (e.g., hypertension, diabetes, hyperlipidemia, and smoking status) among the CSVD-GS, CSVD-PS, and HC groups. Compared with the CSVD-GS and HC groups, the CSVD-PS group displayed higher PSQI and HAMD scores. Both CSVD subgroups demonstrated more severe WMH, EPVS burden, and higher risk of lacunes and CMBs than those in the HC group. In contrast, there were no statistically significant differences in WMH, EPVS burden, lacunes, or CMBs between the CSVD-GS and CSVD-PS groups.

### 3.2. Group Differences in GMV

**Figure 2:**
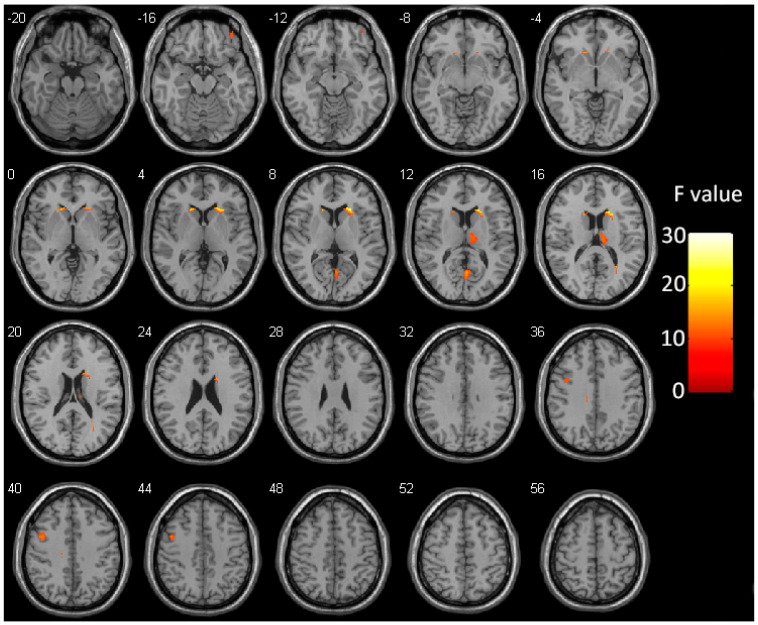
Whole brain group differences in GMV. Color regions represent significant differences in GMV (*p* < 0.05, FDR corrected, cluster size >40 voxels). The color bar represents the corresponding F-values. GMV—gray matter volume; FDR—false-discovery rate.

Results from the one-way ANOVA revealed that the GMV of bilateral caudate, right thalamus, bilateral calcarine cortex, left precentral gyrus, right orbitofrontal cortex, left cingulate gyrus, and right sub-gyral of the temporal lobe differed among the three groups (*p* < 0.05, FDR corrected, cluster size >40 voxels) (Figure 2 and Figure S1 and Table 2).

**Figure 3:**
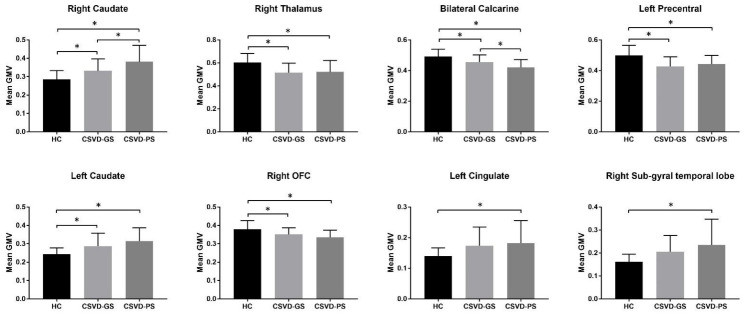
ROI post hoc tests. X-axis represents three groups of subjects; Y-axis indicates the mean GMV of each ROI. * Significant at *p* < 0.05 (Bonferroni corrected). ROI—region of interest; GMV—gray matter volume; OFC—orbitofrontal cortex; CSVD—cerebral small vessel disease; HC—healthy controls; GS—good sleepers; PS—poor sleepers.

Among brain regions that showed significant GMV differences among the three groups, post-hoc tests were completed to further determine group differences. The GMV of the right thalamus, bilateral calcarine cortex, left precentral gyrus, and right orbitofrontal cortex in the CSVD-GS group was significantly decreased compared to the HC group. Further, the GMV of the bilateral caudate was significantly increased in the CSVD-GS group compared to the HC group. In addition to the above alterations, when compared with the HC group, the CSVD-PS group also showed increased GMV in the left cingulate gyrus and right sub-gyral of the temporal lobe. Finally, the CSVD-PS group exhibited decreased GMV in the bilateral calcarine cortex yet increased GMV in the right caudate compared to the CSVD-GS group (Figure 3).

### 3.3. Associations among Sleep Quality, GMV, and WMH in CSVD Patients

Results from the linear regression analyses revealed that WMH volume was positively associated with bilateral caudate, right sub-gyral of the temporal lobe, and left cingulate gyrus GMV and negatively correlated with mean GMV of the bilateral calcarine cortex in CSVD patients (Supplementary Table S1). Additionally, there was a positive correlation between PSQI score and WMH volume (β = 0.383, 95% CI 0.110 to 0.656, *p* = 0.007). Adjusting for depressive symptoms did not attenuate these associations.

In CSVD patients, the overall PSQI score was positively associated with the mean GMV of the bilateral caudate and right sub-gyral of the temporal lobe and negatively associated with the mean GMV of the bilateral calcarine cortex after adjusting for age and sex. However, the association between the PSQI score and mean GMV of the right sub-gyral of the temporal lobe was no longer significant after adjusting for depressive symptoms (model 2) (Table 3). In the fully adjusted model (model 3), the PSQI score was positively associated with the mean GMV of the right caudate and negatively associated with the mean GMV of the bilateral calcarine cortex (Table 3).

## 4. Discussion

In the present study, we performed an exploratory whole-brain VBM analysis to investigate structural brain differences in CSVD patients with and without sleep disturbance. Results showed that both CSVD groups exhibited structural brain alterations compared to the HC group. And compared to the CSVD-GS group, the CSVD-PS group showed significantly reduced GMV in the bilateral calcarine cortex and increased GMV in the right caudate. In addition, the mean GMV of the right caudate and bilateral calcarine cortex were both associated with the total PSQI score in CSVD patients.

The current study demonstrated that CSVD patients exhibited gray matter alterations in cortical (frontal lobe, inferior temporal lobe, occipital lobe, and cingulate gyrus) and subcortical (thalamus and caudate) regions compared with the HC. Similar to our results, the decreased GMV in the frontal lobe and thalamus has been observed in several other neuroimaging studies among CSVD patients [22,23,24]. The frontal lobe and thalamus are a part of the salience network and play an important role in cognitive processes. Therefore, the decreased GMV of the frontal lobe and thalamus may be associated with cognitive impairment in CSVD patients [22]. Additionally, previous neuroimaging studies examining CSVD patients with cognitive impairment or gait disorders also found increased GMV and functional connectivity within the caudate, suggesting that the caudate may be involved in motor disturbance in CSVD patients [22,25]. Although our study showed that brain structure differed between the CSVD groups and the HC group, when comparing brain structure between the CSVD-GS group and CSVD-PS group, differences were only found in the right caudate and bilateral calcarine gyrus, indicating that these two regions may underlie sleep disturbance in CSVD patients.

We identified significantly increased GMV of the right caudate in CSVD patients with poor sleep quality compared to CSVD patients with good sleep quality. GMV of the right caudate was positively associated with sleep quality severity. The caudate nucleus is a main component of the corpus striatum and, together with the putamen, forms the dorsal striatum [26]. The dorsal striatum is a major input pathway of the basal ganglia and is involved in sleep-wake regulation [27]. Experimental studies using animal models have shown that caudate lesions in cats induce restlessness and hyperreactivity [28]. Further, among inpatients with intractable epilepsy, electrical stimulation of the caudate affects cortical excitability [29]. Similar to our results, two previous studies found that the increased GMV of the caudate was associated with sleep disturbance in healthy participants [30,31]. Diffusion tensor imaging (DTI) studies have revealed that patients with primary insomnia show alterations in topological properties of the caudate and reduced caudal connectivity with frontal regions [32,33,34]. A resting-state functional MRI study found that patients with insomnia showed increased resting-state functional connectivity between the left nucleus accumbens and bilateral caudate [35]. In the present study, we found that increased caudate nucleus volume among CSVD patients with poor sleep quality may suggest the presence of neuroplastic compensation that prevents disease progression. Further investigations using a longitudinal design are necessary to determine whether the compensatory mechanisms eventually give way to decompensation during the disease progression.

Additionally, we also found that GMV of the bilateral calcarine cortex was decreased in the CSVD-PS group compared to the CSVD-GS group. Further analysis revealed a negative correlation between the GMV of the bilateral calcarine cortex and PSQI score among CSVD patients. The calcarine cortex is also known as the primary visual cortex, which is located near the calcarine fissure of the occipital lobe and is involved in processing visual information [36]. Insomnia is considered to be a state of hyperarousal [37]. Structural and functional alterations in sensory brain regions (including the area of the visual cortex) of patients with insomnia have been previously reported using MRI [38]. Tao et al. suggested that the decreased functional connectivity of the left suprachiasmatic nuclei with the right lingual gyrus and left calcarine sulcus was correlated with early-wakening symptoms in patients with major depressive disorder [39]. Gamma-aminobutyric acid (GABA) is the main inhibitory neurotransmitter in the brain and plays an important role in sleep regulation [40]. Previous studies have reported that reduced GABA in the occipital cortex is associated with hyperarousal in insomnia patients [40,41]. Based on previous findings, we propose that damage to brain regions related to visual processing may be a potential structural basis for the comorbidity of CSVD and sleep disturbance.

In the current study, GMV of the sub-gyral temporal lobe was associated with sleep disturbance severity, but the association disappeared after controlling for depression. Among older adult populations, sleep disturbance and depression are highly comorbid [42]. Therefore, we included depression as a potential confounder. Although the temporal lobe is thought to be primarily involved with cognition and emotion regulation [43,44], several studies have found both structural and functional abnormalities of the temporal lobe among patients with primary insomnia [45,46,47,48]. Li et al. found that both patients with subthreshold and clinically diagnosed insomnia exhibited increased GMV of the right middle and inferior temporal gyrus relative to healthy controls and that GMV was correlated with emotional score [48]. Our findings suggest that the association between sleep quality and temporal lobe volume may be mediated by sleep-dependent emotional processing.

Although our findings show an association between sleep disturbance severity and GMV alterations in CSVD patients, the cross-sectional study design limits inferences of causation. The relationship between sleep disturbance and brain alterations is complex and bidirectional. Sleep disturbance might aggravate the CSVD burden and vice versa. For instance, some basic experiments have demonstrated that sleep facilitates the proliferation of oligodendrocyte precursor cells and myelination [49,50]. Furthermore, shorter sleep duration influences the microstructural integrity of white matter by impairing oligodendrocyte function [51,52]. Additionally, sleep disturbance is related to many pathophysiological processes, such as neuroinflammation, metabolite clearance, and oxidative stress, which may affect brain structure [53,54,55]. Further, WMH and lacunes may contribute to sleep disturbance through the destruction of sleep-related cortical-subcortical circuits in CSVD patients [56,57].

In addition to the cross-sectional nature of this study, other limitations should be acknowledged. First, the sample size is relatively small, which may limit our statistical power. Second, sleep quality was measured using a self-report questionnaire instead of objective measures (e.g., polysomnography), which may have resulted in bias. Finally, in order to improve the diagnostic accuracy of poor sleep quality, borderline cases were excluded from the present study. Overall, the current study provides preliminary findings. Future longitudinal studies with larger sample sizes are required to further explore the mechanisms underlying the comorbidity of CSVD and sleep disturbance.

## 5. Conclusions

In conclusion, the present study revealed structural brain alterations in CSVD patients with sleep disturbance. Findings from the current study further demonstrated a relationship between GMV and sleep quality in CSVD patients. These findings may provide novel insight into the neural mechanisms of sleep disturbance in CSVD.

## Figures and Tables

**Table 1:** Demographic and clinical characteristics.

Characteristics	HC (*n* = 40)	CSVD-GS (*n* = 23)	CSVD-PS (*n* = 36)	Overall *p* Value
Age (years)	61.53 ± 6.07	64.91 ± 6.40	64.31 ± 7.09	0.079
Male, *n* (%)	25 (62.5%)	15 (65.2%)	16 (44.4%)	0.180
Education (years)	9.73 ± 3.87	9.65 ± 4.58	9.03 ± 4.16	0.740
Hypertension, *n* (%)	17 (42.5%)	14 (60.9%)	23 (63.9%)	0.137
Diabetes, *n* (%)	7 (17.5%)	5 (21.7%)	8 (22.2%)	0.854
Hyperlipidemia, *n* (%)	8 (20.0%)	8 (34.8%)	12 (33.3%)	0.319
Smoking status, *n* (%)	11 (27.5%)	7 (30.4%)	8 (22.2%)	0.840
PSQI	3.88 ± 1.28	3.61 ± 1.16	12.36 ± 2.97	<0.001 ^a,b^
HAMD	4.60 ± 2.81	3.43 ± 3.22	6 ± 2.11	0.002 ^a,b^
TIV (cm3)	1524.14 ± 138.74	1528.61 ± 115.41	1520.06 ± 160.02	0.975
Total Fazekas score	1.08 ± 0.83	4.39 ± 1.12	4.64 ± 1.20	<0.001 ^a,c^
Total WMH volume (cm3)	1.12 ± 1.37	15.99 ± 4.51	22.10 ± 22.63	<0.001 ^a,c^
Lacunes, *n* (%)	0	6 (26.1%)	10 (27.8%)	<0.001 ^a,c^
Microbleeds, *n* (%)	0	8 (34.8%)	14 (38.9%)	<0.001 ^a,c^
>10 EPVS_BG, *n* (%)	2 (5.0%)	8 (34.8%)	15 (41.7%)	<0.001 ^a,c^

^a^ Significant difference between the CSVD-PS and HC groups. ^b^ Significant difference between the CSVD-PS and CSVD-GS groups. ^c^ Significant difference between the CSVD-GS and HC groups. CSVD—cerebral small vessel disease; HC—healthy controls; GS—good sleepers; PS—poor sleepers; PSQI—Pittsburgh Sleep Quality Index; HAMD—Hamilton Depression Rating Scale; TIV—total intracranial volume; WMH—white matter hyperintensities; EPVS—enlarged perivascular spaces; BG—basal ganglia.

**Table 2:** GMV differences among groups.

Brain Region	Hemisphere	Cluster Size	MNI Coordinates			Peak F Values
			X	Y	Z	
Caudate	R	387	21	22.5	10.5	28.14
Thalamus	R	282	9	−16.5	15	12.24
Calcarine cortex	Bilateral	198	1.5	−64.5	10.5	15.34
Precentral gyrus	L	177	−46.5	4.5	42	12.84
Caudate	L	148	−18	24	4.5	21.13
Orbitofrontal cortex	R	61	37.5	45	−18	11.16
Cingulate gyrus	L	58	−12	−24	33	12.57
Sub-gyral of the temporal lobe	R	45	27	−55.5	18	16.86

GMV—gray matter volume; L—left; R—right; MNI—Montreal Neurological Institute.

**Table 3:** Association between PSQI score and mean gray matter volume in CSVD patients.

Independent Variable	Model 1		Model 2		Model 3	
	β (95% CI)	*p*	β (95% CI)	*p*	β (95% CI)	*p*
Right Caudate	0.399 (0.147; 0.650)	0.002	0.362 (0.128; 0.595)	0.003	0.315 (0.048; 0.583)	0.022
Right Thalamus	−0.156 (−0.449; 0.137)	0.290	−0.075 (−0.352; 0.202)	0.588	−0.045 (−0.317; 0.227)	0.740
Bilateral Calcarine cortex	−0.421 (−0.672; −0.171)	0.001	−0.397 (−0.627; −0.167)	0.001	−0.354 (−0.598; −0.109)	0.005
Left Precentral gyrus	0.240 (−0.043; 0.523)	0.095	0.232 (−0.028; 0.492)	0.079	0.216 (−0.038; 0.470)	0.094
Left Caudate	0.301 (0.039; 0.564)	0.025	0.260 (0.015; 0.505)	0.038	0.187 (−0.084; 0.457)	0.172
Right Orbitofrontal cortex	−0.089 (−0.383; 0.204)	0.544	−0.064 (−0.335; 0.207)	0.638	−0.086 (−0.351; 0.178)	0.517
Left Cingulate gyrus	0.321 (−0.009; 0.650)	0.056	0.282 (−0.023; 0.588)	0.069	0.161 (−0.204; 0.526)	0.380
Right Sub-gyral of the temporal lobe	0.399 (0.094; 0.704)	0.011	0.261 (−0.050; 0.571)	0.098	0.142 (−0.210; 0.495)	0.421

Model 1: adjusted for age and sex. Model 2: adjusted for age, sex, and depressive symptoms. Model 3: adjusted for age, sex, depressive symptoms, and WMH volume. PSQI—Pittsburgh Sleep Quality Index; CSVD—cerebral small vessel disease; CI—confidence interval.

## Data Availability

The data that support the findings of this study are available from the corresponding author upon reasonable request.

## Supplementary Materials

The following supporting information can be downloaded at: https://www.mdpi.com/article/10.3390/brainsci13020294/s1, Figure S1: Whole brain group differences in GMV (sagittal view); Table S1: Association between WMH volume and abnormal mean gray matter volume in CSVD patients.
