# Supplementary material for: Differences in Gray Matter Volume in Cerebral Small Vessel Disease Patients with and without Sleep Disturbance
Source: Brain Sci. 2023 Feb 9;13(2):294. doi: 10.3390/brainsci13020294 (PMC9953873; doi:10.3390/brainsci13020294)
Supplement: Supplementary file 1 [file brainsci-13-00294-s001.zip › brainsci-2141622-supplementary.pdf]

Supplementary Material

Supplementary Figure S1. Whole brain group differences in GMV (sagittal view).

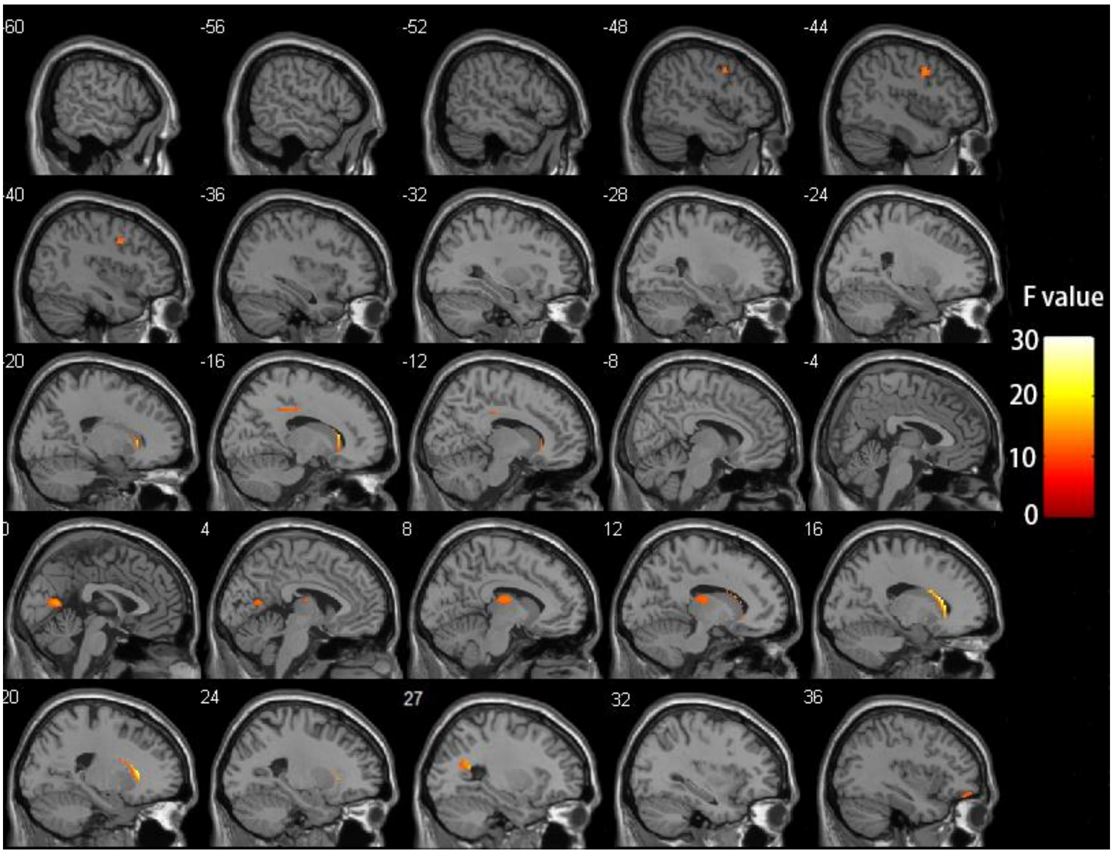

Supplementary Table S1. Association between WMH volume and abnormal mean gray matter volume in CSVD patients

Dependent variable	Model 1		Model 2	
	β (95%CI)	P	β (95%CI)	P
Right Caudate	0.518 (0.266;0.769)	**<0.001**	0.535 (0.269;0.801)	**<0.001**
Right Thalamus	−0.159 (−0.422;0.103)	0.229	−0.114 (−0.389;0.161)	0.408
Bilateral Calcarine cortex	−0.362 (−0.631; −0.092)	**0.009**	−0.376 (−0.661; −0.091)	**0.011**
Left Precentral gyrus	0.068 (−0.203;0.339)	0.616	0.069 (−0.218;0.355)	0.632
Left Caudate	0.469 (0.213;0.726)	**0.001**	0.478 (0.206;0.750)	**0.001**

Right Orbitofrontal cortex	0.059 (−0.208;0.326)	0.659	0.084 (−0.198;0.366)	0.553
Left Cingulate gyrus	0.470 (0.277;0.664)	**<0.001**	0.495 (0.291;0.698)	**<0.001**
Right Sub-gyral of the temporal lobe	0.488 (0.283;0.693)	**<0.001**	0.433 (0.222;0.643)	**<0.001**

Model 1: adjusted for age and sex. Model 2: adjusted for age, sex, and depressive symptoms. WMH, white matter hyperintensities; CSVD, cerebral small vessel disease; CI, confidence interval.
